# Effect of Ground Transportation on Adrenocortical Activity in Prepuberal Female Mice from Five Different Genetic Backgrounds

**DOI:** 10.3390/ani9050239

**Published:** 2019-05-15

**Authors:** Sonja Rumpel, Christina Scholl, Anja Göbel, Rupert Palme, Esther Mahabir

**Affiliations:** 1 Charles River Laboratories, Sandhofer Weg 7, 97633 Sulzfeld, Germany; Sonja.Rumpel@crl.com (S.R.); Christina.Scholl@crl.com (C.S.); Anja.Goebel@crl.com (A.G.); 2 Department of Biomedical Sciences, University of Veterinary Medicine, Veterinärplatz 1, A-1210 Vienna, Austria; Rupert.Palme@vetmeduni.ac.at; 3 Comparative Medicine, Center for Molecular Medicine, University of Cologne, Robert-Koch-Straße 21, 50931 Cologne, Germany

**Keywords:** mouse, transportation, stress, glucocorticoids, animal welfare

## Abstract

**Simple Summary:**

For research purposes, mice are often transported between institutions, which may elicit stress, thereby influencing results. We determined adrenocortical activity by measuring fecal corticosterone metabolites (FCMs), as a stress marker, in prepuberal mice from five genetic backgrounds, namely C57BL/6J, C57BL/6NCrl, FVB/NCrl, Crl:CD1(ICR), and BALB/cAnCrl. Only C57BL/6N showed significantly higher FCM levels the day after transport, but baseline levels were attained within four days.

**Abstract:**

Specific experimental protocols necessitate transportation, a potentially stressful event that could confound results. We determined adrenocortical activity by measuring fecal corticosterone metabolites (FCMs), as a stress marker, in prepuberal (three-week old) female C57BL/6J, C57BL/6NCrl, FVB/NCrl, Crl:CD1(ICR), and BALB/cAnCrl mice. On each transport day, five female cage mates per genetic background were weaned and transported in stable groups via truck from the breeding to the research facility. Fecal pellets were collected on Days 0, 1, and 4. Mice were superovulated for embryo production to determine if repeated fecal collection impacts this procedure. The average duration of transportation over 600 km and from packing to unpacking of mice was 7.24 and 22.62 h, respectively. FCM levels increased from Day 0 to Day 1 and decreased on Day 4 in all genetic backgrounds except in FVB/NCrl, but only B6N showed significantly higher FCM levels on Day 1. Furthermore, embryo production was not affected by repeated feces collection. The results show that weaning and immediate transport of prepuberal mice from the breeding to the research facility led to temporal and genetic background-dependent increases of adrenocortical activity in four of the five genetic backgrounds investigated, which returned to baseline levels within four days.

## 1. Introduction

Concomitant with the high demand for genetically engineered mice for biomedical research, transportation of mice or their genetic material in the form of oocytes, embryos, or spermatozoa is indispensable. Transportation of live mice is executed either by air and/or via ground transportation. Moreover, specific experimental protocols such as superovulation of oocyte or embryo donors necessitate transportation of prepuberal mice immediately after weaning. When weaned by commercial suppliers, mice are separated from their cage mates and dam and may even be re-grouped with animals from other cages. During transportation and upon receipt, they are exposed further to a different environment, which may include vibrations, sounds, disruption of the dark/light regime, temperature, humidity, feed, bedding, water, odors, unfamiliar animal caretakers, and a new social structure. These factors may induce stress as a cumulative burden, possibly affecting animal welfare and confounding research results. Therefore, an adaptation period to the new housing conditions is recommended [1]. To evaluate stress in animals, a number of parameters can be used. One of these is the measurement of fecal corticosterone metabolites (FCMs) since corticosterone is the major glucocorticoid in mice and its metabolites are excreted primarily via feces [2,3]. Thus, this approach is non-invasive and does not elicit a stress response, in contrast to invasive methods such as blood sampling [4]. In the few reports available on transportation of mice, body weight and immune responses decreased while plasma corticosterone levels increased up to at least 48 h after 18–48 h of transport [5,6,7,8].

For assisted reproductive technological work at the Center for Molecular Medicine, University of Cologne (CMMC), prepuberal female mice are imported from the commercial supplier and transported overnight over a distance of approximately 600 km. The hormone administration for superovulation begins three days later. Since the degree and time of disturbance of homeostasis after transportation of prepuberal mice is unknown, we non-invasively determined induced stress to the animals by measuring adrenocortical activity over a five-day period in mice of five different genetic backgrounds that were transported from the breeding to the research facility. Furthermore, mice were superovulated and two-cell embryos were collected to determine if additional handling of mice due to repeated collection of fecal samples affects embryo production.

## 2. Animals, Materials, and Methods

### 2.1. Mice

Female inbred C57BL/6J (B6J), C57BL/6NCrl (B6N), BALB/cAnCrl (BALB/cN), and outbred Crl:CD1(ICR) (CD-1) mice were weaned at 21 days of age. Outbred FVB/NCrl (FVB/N) mice were weaned at 21 to 24 days. On each day of transport, five female cage mates from each genetic background were weaned together and then kept in stable groups throughout the study. At both facilities, mice were kept under standard husbandry conditions according to the Directive 2010/63/EU and were free from the Federation of European Laboratory Animal Science Associations (FELASA)-listed infectious agents.

### 2.2. Breeding and Husbandry at Charles River Laboratories

At Charles River Laboratories (CRL), mice were born and raised in three different barrier facilities (B6J: Barrier 1; B6N, BALB/cN, and FVB/N: Barrier 2, and CD-1: Barrier 3). They were kept in Type III open-top cages with autoclaved bedding (Ecopure7D, Datesand group, Manchester, UK) and paper tissue as nesting material at a temperature of 21 °C ± 0.5 °C, humidity of 50 ± 20%, an average of 16 air changes per hour in the room, and a 12/12-hour light/dark cycle (lights on at 6:00 a.m.). Mice were fed a standardized autoclaved pelleted mouse diet (VRF1 (P), SDS, Witham, Essex, UK) and given sterilized drinking water ad libitum.

In mice from all genetic backgrounds, harem matings were practiced with cross-fostering of 20 to 50 pups per three to five dams. In the present study, for each genetic background, five prepuberal female mice from the same breeding cage were randomly selected and separated from their dams and kept in Type III cages for up to 1 h during which voided fecal pellets were collected (Day 0). The work done in Barriers 1, 2, and 3 at CRL was performed by three, one, and three different caretakers, respectively. They were aware of the genetic background of the mice they were handling.

### 2.3. Transport of Mice

**Figure 1:**
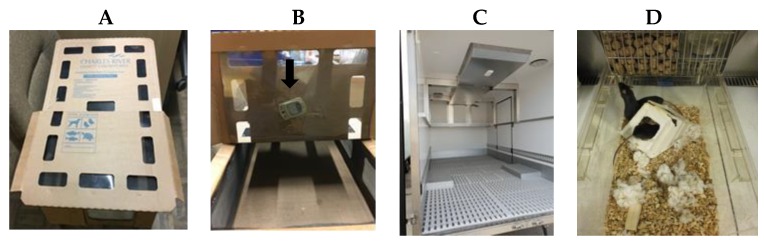
(**A**) Transport crate; (**B**) data logger inside of crate (arrow); (**C**) inside of truck; (**D**) holding cage with enrichment.

After fecal pellet collection, each group of mice was packed in a filtered transportation crate (R/M-Karton, 630 × 420 × 170 mm, Krug, Bad Königshofen, Figure 1A), which contained bedding (Ecopure7D, Datesand group, Manchester, UK), nesting material (Zellstoffwatte, LMS Consult, Brigachtal, Germany), feed (VRF1 (P), SDS, Witham, Essex, UK), and a source of water (Hydrogel, Clear H_2_O, Portland, OR, USA). A data logger (EBI 20-TH1 Xylem Analytics, Germany Sales GmbH & Co. KG, Ingolstadt, Germany) measuring temperature and relative humidity from packing to unpacking of mice was inserted in each crate (Figure 1B).

Immediately thereafter, mice were transported from CRL, Sulzfeld to the CMMC research facility in air-conditioned vehicles (Figure 1C) but were not exposed to light during transport. The different steps involved in ground transportation are shown in Table 1. Upon arrival at the CMMC building (Day 1), mice were kept in their crates in a heating cabinet (Allentown, NJ, USA) at 20 to 24 °C because the technicians work in the laboratories in the CMMC building first. Afterward, the technicians transported the crates with the mice manually (held in the hands during a 2-min walk) to the quarantine of the CMMC, which is situated in a neighboring building. Both the heating cabinet and the quarantine are on ground level.

Mice from each genetic background were transported on six different days in the period from February to April 2018. A total of 30 female mice from each of the genetic backgrounds (B6J, B6N, FVB/N, CD-1, and BALB/cN) were used in the present study.

### 2.4. Mouse Husbandry in the CMMC Quarantine

In the CMMC quarantine, each group of five mice was kept in Type II long individually ventilated cages (IVCs, Greenlines, Tecniplast, Buggugiate, Italy) with bedding (FS14, J. Rettenmaier and Söhne, Rosenberg, Germany), houses (Mouse Smart Homes, Datesand group, Manchester, UK), wooden gnawing sticks (J. Rettenmaier and Söhne), and nestlets (Arbocell, Rettenmaier and Söhne) (Figure 1D). They were kept at 20 to 24 °C, 50 to 70% relative humidity, 75 air changes per hour in the cages, and a 12/12-hour light/dark cycle (lights on at 6:00 a.m.). Mice were fed an autoclaved standardized mouse diet (1314P, Altromin, Lage, Germany) and given autoclaved drinking water ad libitum. No animal care activities were performed after mice were placed in their home cage on Day 1 to exclude confounding factors. The same two technicians performed all steps throughout the study. They were aware of the genetic background of the mice they were handling.

### 2.5. Fecal Pellet Collection 

Voided fecal pellets were collected from the groups of mice temporarily kept on paper towels without bedding between 1:00 p.m. and 2:15 p.m. on Day 0 (at CRL) to obtain baseline values of stress hormones secreted 8 to 10 h before [3]. Feces were also collected at the same time on Day 1 and Day 4 (day of first hormone treatment described later) at the CMMC quarantine facility 24 h and 96 h later, respectively, to eliminate any influences of the circadian rhythm on hormone secretion. Samples were collected in microcentrifuge tubes and stored at −20 °C until analysis. To simulate routine conditions whereby mice are not handled after arrival at the CMMC quarantine until Day 4, fecal pellets were not collected on Day 2 and Day 3.

### 2.6. Analysis of Fecal Corticosterone Metabolites

Fecal samples were dried in an oven at 70 °C for 24 h, homogenized using a mortar and pestle to form a powder, and weighed. An amount of 50 mg was shaken with 1 mL of 80% methanol in an Eppendorf tube for 30 min on a vortex and centrifuged at 2500× *g* for 15 min. If this amount of fecal pellets was not available after 1 h of collection, the amount was shaken with the corresponding amount of methanol (e.g., 40 mg of feces with 0.8 mL of methanol). The supernatant was transferred into a new Eppendorf tube and stored at −20 °C until analysis. FCMs were determined using a well-established 5α-pregnane-3β,11β,21-triol-20-one enzyme immunoassay (EIA) [2,3]. In total, 90 samples were analyzed—six groups of mice from each of the five genetic backgrounds with fecal samples being collected on three different days (Day 0, Day1, and Day 4).

### 2.7. Superovulation and in vivo Production of Two-Cell Embryos

On Day 4, immediately after fecal pellet collection, females were induced to superovulate with an intraperitoneal injection of 5 IU equine chorionic gonadotropin (eCG, PMSG, Cat-Nr.: OPPA01037, Hölzel Diagnostika Handels GmbH, Cologne, Germany) and an intraperitoneal injection of 5 IU human chorionic gonadotropin (hCG, Ovogest, Cat-Nr: 707184, Intervet, Unterschleissheim, Germany) given 48 h apart between 2:00 p.m. and 3:00 p.m. Immediately after the hCG injection (Day 6), females were paired with males, which were at least 12 weeks old. After cervical dislocation of the females on Day 8, Day 1.5 embryos (two-cell) were flushed from the excised oviducts of all females using M2 medium (Sigma), as previously described [9]. Morphologically intact two-cell embryos with two blastomeres of approximately the same size, homogeneous cytoplasm, intact zonae pellucida, and neither blebbing nor fragmenting were selected using a stereo microscope (Zeiss, Jena, Germany) under 400× magnification. For each group of five mice, embryos were collected in the same petri dish.

### 2.8. Animal Welfare

B6J, B6N, FVB/N, CD-1, and BALB/cN were used in the present study because most genetically modified lines in the CMMC facility are kept on these genetic backgrounds. Mice from these genetic backgrounds which are bred in-house at the CMMC are re-stocked every two years with embryo donors from approved sources to prevent genetic drift. Only animals used in this context were evaluated. As such, the work described here did not warrant a special license, as it was in compliance with the German Animal Welfare Act, and met the standards of the Animal Research: Reporting In Vivo Experiments (ARRIVE) guidelines [10]. Furthermore, the same technicians performed all steps at the CMMC quarantine throughout the study. Non-invasive sample collection, as well as group-housing, was implemented throughout the study to reduce stress and to enhance animal welfare.

### 2.9. Statistical Analysis

FCM data are presented as means ± standard error of the mean (SEM)/50 mg of feces. Differences in FCM were analyzed using a two-way ANOVA (for day and genetic background) and post hoc pair-wise *t*-tests. The numbers of embryos per group of five females from the five different genetic backgrounds were compared employing the Kruskal–Wallis Test, which is a robust non-parametric one-way ANOVA. On that basis, *p*-values  ≤  0.05 were considered statistically significant. The statistical programming software package SAS 9.4 was employed (SAS Institute Inc: SAS/STAT User’s Guide, Version 9.4, SAS Institute Inc.: Cary, NC, USA, 2014).

## 3. Result

### 3.1. Transportation Duration

Table 1 shows the different stages of the transportation process from the breeding to the quarantine facility in the chronological order in which they were performed. The average duration of transport was 7.24 ± 0.19 h, while the average time from packing of mice at the breeding facility to unpacking of mice in the quarantine facility was 22.62 ± 0.07 h. Transport to the CMMC research facility is not direct and includes the mandatory overnight rest for the driver and at least one other stop prior to delivery at the CMMC, which lengthens the transport duration. In the crates, an average temperature of 20 °C and 40 to 50% relative humidity were measured during transport. All mice were in good physical condition upon arrival and throughout the husbandry period.

### 3.2. Feces Excretion

The amount of feces (dry weight in mg) excreted per group within a maximum of 1 h of collection according to genetic background is shown in Table 2. At least 20 fecal pellets were collected within this period from each group of mice, which were generally sufficient for FCM determination. There were only three samples where less than 50 mg of ground fecal sample was available (35, 40, 40 mg), but we adjusted the amount of methanol used for extraction accordingly.

### 3.3. Fecal Corticosterone Metabolites (FCMs)

**Figure 2:**
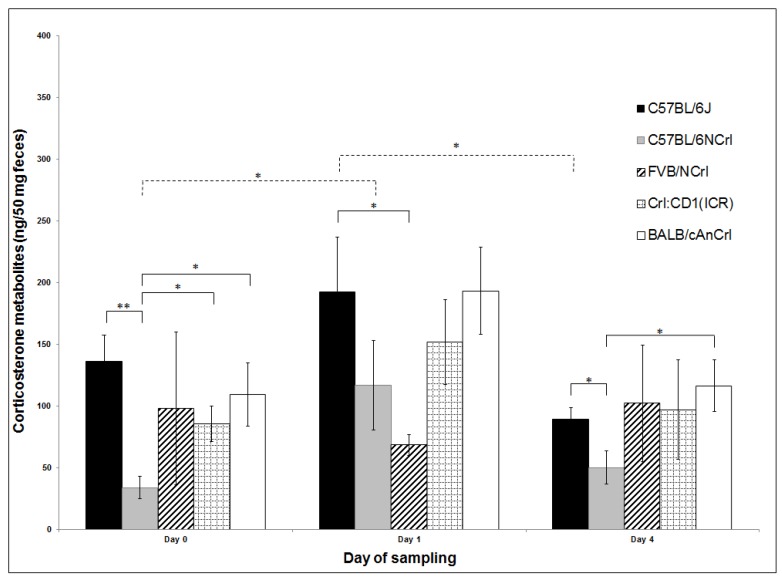
Concentrations of fecal corticosterone metabolites (ng/50 mg of feces; mean ± standard error of the mean (SEM)) according to genetic background and day of transport in prepuberal female mice after ground transportation; * *p* < 0.05, ** *p* < 0.01.

Levels of corticosterone metabolites (ng/50 mg of feces) are shown in Figure 2. The two-way ANOVA of FCM concentrations revealed significant differences between days (*p* = 0.0138) and genetic backgrounds (*p* = 0.0259). FCM levels increased from Day 0 to Day 1 and decreased on Day 4 in all genetic backgrounds except in FVB/N, where values were lowest on Day 1 (68.7 ± 8.4 ng/50 mg of feces). However, significant higher FCM levels on Day 1 compared to Day 0 were observed only in the B6N strain. Compared to Day 4, FCM levels in the B6J strain were significantly higher on Day 1. Among genetic backgrounds, on Day 0, levels were significantly lower in B6N (34 ± 9.1 ng/50 mg) compared to B6J (136.2 ± 21.1 ng/50 mg), CD-1 (85.5 ± 14.4 ng/50 mg), and BALB/cN (109.3 ± 25.6 ng/50 mg). Significantly lower levels were observed for FVB/N (68.7 ± 8.4 ng/50 mg) compared to B6J (192.7 ± 44.6 ng/50 mg) on Day 1, and for B6N (50.3 ± 13.4 ng/50 mg) compared to B6J (89.7 ± 8.9 ng/50 mg) and BALBc/N (116.6 ± 21.1 ng/50 mg) on Day 4.

### 3.4. Embryo Production

The prepuberal mice were superovulated and two-cell embryos were collected to determine whether additional handling due to fecal collection had an effect on embryo production. Also, the efficacy of the number of embryos produced to that normally obtained in our laboratory [11] was compared. The number of two-cell embryos collected is shown in Table 3.

There were no significant differences between genetic backgrounds with respect to the number of embryos collected (*p* < 0.2749).

## 4. Discussion

This is the first study to evaluate the effect of ground transportation on adrenocortical activity in prepuberal female mice from five different genetic backgrounds that are commonly employed in biomedical research. The present results show that transportation of prepuberal mice from the breeding to the research facility led to increased FCM levels on Day 1 in four out of five genetic backgrounds, and baseline levels were attained within four days. However, only B6N showed significantly higher FCM levels on Day 1. On any of the three days investigated, FCM levels differed according to genetic background. Furthermore, embryo production was not affected by handling due to repeated fecal collection.

There are indeed sparse reports pertaining to the effects of transportation on mice [1]. The transportation mode can be in-house, domestic, or international, with the latter often being longer. As such, the type and duration of transport may affect corticosterone metabolite levels, which may, in turn, influence the time required to return to baseline levels. Drozdowicz et al. [7] reported that a 12-min in-house transfer of 8–12-week-old male BALB/cAnNCrl(BR) led to increased plasma corticosterone over the following 24 h. The report by Tuli et al. [12] showed that a 12-min in-house transport (10-min walk and 2 min in an elevator) of inbred 4.5–5-month-old male BALB/c/Ola mice led to a significant elevation in serum corticosterone levels immediately after transportation, but baseline levels were attained within one day. However, they reported that behavioral activity was still altered after four days. Serum corticosterone concentrations were elevated in 4–5-week-old C57BL/6 and ICR mice after 3–4 h of transportation by truck [13]. Plasma corticosterone levels increased and immune function decreased up to at least 48 h after 36–48 h of transport by truck or a 24–36 h of transport by plane in eight-week-old Crl:COBS,CD-1(ICR)BR mice as reported by Landi et al. [5]. Aguila reported that transport of six-week-old female C57BL/6J mice by air-conditioned trucks (36 to 42 h) or by air (18 to 20 h) led to decreases in natural killer cell activity and increases in corticosterone levels, but mice were acclimatized after 24 h [6]. The abovementioned studies measured corticosterone levels using radioimmunoassay.

Sex was reported to influence corticosterone metabolism and FCM levels since higher levels were measured in females compared with their male counterparts [2,3,14]. As such, it is expected that FCM levels in prepuberal male mice from the genetic backgrounds used in this study will also be lower than those observed for the female mice used. Furthermore, the genetic background and the age of mice also have a significant effect on the level of stress hormone metabolites, as demonstrated for female mice from an inbred (C57BL/6NCrl) and an outbred stock (Crl:CD1), which were born and bred in-house up to the age of 26 months [15]. The latter researchers showed that corticosterone levels increased with age in Crl:CD1 mice, whereas levels were relatively constant in C57BL/6NCrl mice, which may reflect differences in adrenocortical activity or different corticosterone metabolism [15]. Higher basal serum corticosterone levels were observed in non-transported C57BL/6 compared to ICR mice [13]. The latter observation is in contrast to our results where FCM levels for non-transported prepuberal B6N mice were lower than those for the other four genetic backgrounds. In transported mice, higher increases in serum corticosterone levels were observed for ICR than for C57BL/6 mice compared to their non-transported counterparts [13]. However, this result was not confirmed in our study. Unexpectedly, in the present study, the FCM levels in FVB/N mice decreased on Day 1, in contrast to observations for the other four genetic backgrounds investigated, but differences in FVB/N mice were not significant. The reason for this decrease is unknown, but this may reflect a higher resilience of the FVB/N strain to stress. The present study was performed with prepuberal wild-type mice. Since an increasing number of mice used for biomedical research are genetically engineered and are of different ages, responses to transportation may deviate from those observed in the present study.

During transportation, it is necessary to protect animals from inclement weather and adverse changes in climatic conditions. Mice are kept at 20 to 24 °C in the breeding and research facilities. Therefore, the temperature during transportation should ideally not deviate significantly from these at least for longer periods. In the present study, the temperature in the delivery truck enabled an average of 20 °C in the transportation crate. The time for ground transitions from the breeding facility to the truck and from the truck or holding site after arrival at the research facility, that is, for loading and unloading, should be kept to a minimum since these phases are at greatest risks for temperature deviations. Temperature variations were observed during air shipments of mice [16]. In the present study, the coldest temperature (10.7 to 12.7 °C) experienced from the breeding facility to the truck was for 10 min with two shipments. Between the heating cabinet and the quarantine facility, the temperature was 20 to 22.3 °C for 10 min. Therefore, all temperatures were within the range of 5 to 34 °C for safe transportation [17] and, moreover, in conformity with standard husbandry conditions after loading until unpacking.

Group size did not significantly affect FCM levels [14]. Notably, in the present study, collection of fecal samples from group-housed instead of singly kept mice mimicked routine procedures, did not elicit social stress due to single housing, and allowed determination of FCMs. Therefore, collection of fecal samples, in contrast to blood, is non-invasive and contributes to improved animal welfare [4,18]. Furthermore, this task can be performed easily without any special training.

In the present study, embryo production by the prepuberal female mice was in conformity with previous reports from routine work in our laboratory [11] for which mice are also bought from the same supplier. This implies that feces collection after transfer of mice to clean cages for 1 h on the days reported in this study did not affect embryo production.

Whereas mice were kept in open-top cages at the commercial breeder, they were kept in IVCs in the CMMC quarantine. Previous reports showed that exploratory behavior was not significantly different when group-housed C57BL/6JRj male mice (3–4 mice per cage) were kept in IVCs after being housed for 15 days in conventional caging [19]. Furthermore, FCM concentrations were not significantly different in mice housed in open cages or IVCs [20]. Whether a change in caging from open-top at the Charles River Laboratories to IVC at the CMMC quarantine affected the FCM levels in the present study is unknown, but it should be noted that the defecation rate was comparable for each genetic background, as shown in Table 2.

Previous reports showed that diets with a high fat content may lead to reduced defecation rate, whereby the FCM concentrations might get inflated [21]. In the present study, there was an immediate change of diet upon arrival at the CMMC quarantine. However, both diets showed similar nutrient composition, and defecation rate was not altered. Thus, we do not expect that FCM concentrations were affected by this diet change.

FCM concentrations show a circadian rhythm [2]. Mouse husbandry involves the light and the dark phase, whereby mice are more active in the nocturnal phase. Previous reports showed that FCM concentrations peak 10 h and 4 h after the stress stimuli in the light or the dark phase, respectively [3]. In the present study, it is difficult to pinpoint just one specific time point of stress arousal since the stress stimuli span the period from the time of packing to the time of unpacking the mice. Even though locomotor activity may be influenced by eating behavior, in the present study, it did not influence the defecation rate at the time point of feces collection and, ultimately, the FCM concentration. Notably, we collected feces at the same time point on each day of handling to avoid confounding the results [4].

As a general guide, at least seven days of acclimatization is recommended following transport between sites and at least three days between buildings on the same site [22]. However, depending on the severity and duration of the stress experienced and the parameter to be investigated, the period of acclimatization may take several weeks to normalize, as observed for blood pressure (three weeks) [23], as well as corticosterone and immune parameters (three weeks) [24] in BALB/c mice. When transporting mice prior to experimental procedures, researchers should evaluate the effect on the parameters to be investigated to determine an adequate acclimatization period. Since changes in corticosterone levels often indicate pain and distress, independent of the specific parameters in an investigation, altered FCM levels will give an indication as to whether mice are stressed.

Transportation involves a myriad of steps, as shown in Table 1. Also, weaning itself may be a stressful event. Therefore, the corticosterone levels in the mice investigated in the present study may include these confounding factors. Deciphering the contribution of each of these steps would have meant having controls at each of these steps, thereby increasing the number of mice used. Furthermore, the number of littermates would have been a limiting factor. Therefore, we used baseline values from the same mice to circumvent this challenge, thereby contributing to animal welfare. Under routine experimental conditions, mice may be re-grouped after arrival at the research facility and/or prior to experimentation, which may lead to higher levels of FCMs than those reported in our study. Collecting group samples may also have masked significant effects, because individual differences in baseline and stress-induced FCM levels are present [2,3,4]. However, separating mice for fecal collection, as opposed to keeping them in stable social groups, as practiced in our investigation, may also be stressful. In the present study, notably, while using the same groups of mice for our routine procedures, we were also able to obtain information about the fecal corticosterone metabolite levels of the prepuberal mice that were transported.

Further work could include males, mice of different ages, FCM levels in mice that are weaned in the commercial breeding facility but which are not transported, and behavioral welfare indicators, and it could also consider the influence of different periods of the year.

## 5. Conclusions

We showed in the present study that FCM levels increased in four out of five genetic backgrounds, but baseline levels were reached within four days. Notably, only B6N showed significantly higher FCM levels on Day 1. FCM levels also varied according to genetic background, and embryo production was not affected by handling due to repeated fecal collection.

## Figures and Tables

**Table 1:** Duration of transportation of mice from the breeding to the research facility. SEM—standard error of the mean; CMMC—Center for Molecular Medicine, University of Cologne.

Stage of Transportation	Duration in Hours (Mean ± SEM)
	
Loading at Charles River Laboratories	1.98 h ± 0.08
Transport in truck stage 1	4.26 h ± 0.29
Driver break	0.58 h ± 0.06
Transport in truck stage 2	1.51 h ± 0.23
Driver overnight stop	8.80 h ± 0.14
Transport in truck stage 3	1.47 h ± 0.12
Holding in the heating cabinet at CMMC	3.81 h ± 0.11
Time to unpacking in the quarantine facility	0.22 h ± 0.01
Total duration from packing to unpacking	22.62 h ± 0.07
Total transport time (stage 1 + stage 2 + stage 3)	7.24 h ± 0.19
	

**Table 2:** Amount of feces excreted per prepuberal female within 1 h of collection according to genetic background.

Day of Fecal Pellet Collection	Genetic Background of Mice	Fecal Pellets per Mouse (Mean)	Dry Weight (mg) of Fecal Pellets/Mouse
			
0	B6N	4.4	21.3
0	B6J	5.3	17.4
0	FVB/N	4.2	24.8
0	BALB/cN	4.7	23.6
0	CD-1	4.8	24.0
			
1	B6N	4.5	20.6
1	B6J	4.7	20.0
1	FVB/N	4.3	30.9
1	BALB/cN	5.7	20.5
1	CD-1	4.1	25.6
			
4	B6N	5.7	29.0
4	B6J	5.5	30.2
4	FVB/N	5.0	39.7
4	BALB/cN	4.4	24.3
4	CD-1	6.3	31.0
			

**Table 3:** Number of two-cell embryos per group (*n* = 5) of treated prepuberal females from different genetic backgrounds of mice.

Genetic Background of Mice	Number of Two-Cell Embryos per Group (Mean ± SEM)	Number of Two-Cell Embryos per Female (Mean)
		
B6J	83 ± 17	17
B6N	42 ± 14	8
FVB/N	47 ± 6	9
CD-1	60 ± 13	12
BALB/cN	70 ± 19	14
